# Comparison of 2D, 3D high dose and 3D low dose gated myocardial ^82^Rb PET imaging

**DOI:** 10.1186/1471-2385-7-4

**Published:** 2007-10-22

**Authors:** Karin Knešaurek, Josef Machac, Jong Ho Kim

**Affiliations:** 1 Division of Nuclear Medicine, The Mount Sinai Medical Centre, New York, USA

## Abstract

**Background:**

We compared 2D, 3D high dose (HD) and 3D low dose (LD) gated myocardial Rb-82 PET imaging in 16 normal human studies. The main goal in the paper is to evaluate whether the images obtained by a 3D LD studies are still of comparable clinical quality to the images obtained with the 2D HD or 3D HD studies.

**Methods:**

All 2D and 3D HD studies were performed with 2220 MBq of Rb-82. The 3D LD were performed with 740 MBq of Rb-82. A GE Advance PET system was used for acquisition. Polar maps were created and used to calculate noise among (NAS) and within (NWS) the segments in the noise analysis. In addition, the contrast between left ventricular (LV) wall and LV cavity was also analysed. For 13 subjects, ejection fraction (EF) on 2D and 3D studies was calculated using QGS program.

**Results:**

For the H20 reconstruction filter, the mean contrast in mid-ventricular short-axis slice was 0.33 ± 0.06 for 2D studies. The same contrast for the 3D HD studies was 0.38 ± 0.07 and for 3D LD, it was 0.34 ± 0.08. For the 6 volunteers where 3D HD was used, NAS was 3.64*10^-4 ^and NWS was 1.79*10^-2 ^for 2D studies, and NAS was 3.70*10^-4 ^and NWS was 1.85*10^-2 ^for 3D HD studies, respectively. For the other 10 volunteers where 3D LD was used, NAS was 3.85*10^-4 ^and NWS was 1.82*10^-2 ^for the 2D studies, and NAS was 5.58*10^-4 ^and NWS was 1.91*10^-2 ^for the 3D LD studies, respectively. For the sharper H13 filter, the data followed the same pattern, with slightly higher values of contrast and noise. EF values in 2D and 3D were close. The Pearson's correlation coefficient was 0.90. The average difference from 13 subjects was 8.3%.

**Conclusion:**

2D and 3D HD gating Rb-82 PET cardiac studies have similar contrast, ejection fractions and noise levels. 3D LD gating imaging, gave comparable results in terms of contrast, EF and noise to either 2D or 3D HD gating PET imaging. 3D LD PET gated imaging can make Rb-82 PET cardiac imaging more affordable with significantly less radiation exposure to the patients.

## Background

Not long ago [1], we compared 2D with 3D modes in myocardial ^82^Rb PET imaging at rest. Here, we would like to extend the same comparison to gating myocardial ^82^Rb PET imaging at rest. The gating imaging provides additional useful information like ejection fraction (EF) and wall thickening. However, it is more demanding due to loss of counts (e.g. bad beats rejection) and dynamic memory limitations. Due to the short half-life of ^82^Rb (75 s), ^82^Rb PET cardiac images tend to be count-poor. Additional shifting of counts in different hearth cycle phases makes gating ^82^Rb PET myocardial imaging even more challenging than non-gating imaging. Also, ^82^Rb biokinetics, i.e., high blood pool activity approximately 2 min after I.V. injection, combined with ^82^Rb short half-life, requires a careful acquisition protocol in order to obtain images of adequate quality. While 2D and 3D ^18^F – Fluorodeoxyglucose (FDG) imaging can be optimized based on PET system performance characteristics, mostly described by a noise equivalent count (NEC) rate [2], the dynamic ^82^Rb PET cardiac imaging is more complicated. Optimization of ^82^Rb PET imaging requires taking into account ^82^Rb biokinetics and ^82^Rb short half-life, in addition to the PET system performance characteristics.

The main goal in the paper is to evaluate whether the images obtained by a low dose (LD) of 740 MBq (20 mCi) in the 3D myocardial ^82^Rb perfusion gated PET studies are still of comparable clinical quality to the images obtained with the high dose (HD) of 2220 MBq (60 mCi) in 2D and 3D PET ^82^Rb perfusion PET gated studies. The reduction in dose by a factor of three has significance in reducing costs associated with ^82^Rb and the consequent potential of making ^82^Rb perfusion PET myocardial imaging more affordable. Reducing the patient dose by a factor of three also significantly reduces exposure to the patients.

## Methods

All 2D volunteer studies were performed by injecting I.V. 2220 MBq (60mCi) of ^82^Rb. For six volunteers, 3D studies were performed with a high dose (HD) of 2220 MBq of ^82^Rb and for 10 volunteers in the 3D studies, a low dose (LD) of 740 MBq (20 mCi) of ^82^Rb was used. One volunteer participated twice, i.e., was imaged at two separate occasions, in 2D and 3D HD, and 2D and 3D LD studies, respectively. In all studies, i.e., 2D and 3D LD and 3D HD studies, time per frame was 1 minute and total acquisition time after appropriate delay, was 6 minutes. In the 2D studies there was a delay of 2 minutes, in the 3D LD studies there was a delay of 3 minutes, and in the 3D HD studies there was a delay of 5 minutes. The heart cycle was divided in 8 phases in the 2D gated PET studies. In the 3D gated PET studies, 5 phases were used, due to dynamic memory limitations. These human protocols were approved by the Institutional Review Board of our Medical Center.

The GE ADVANCE (General Electric Medical Systems, Milwaukee, WI) system was used for all acquisitions in both the 2D and 3D modes. The 2D images were reconstructed using a filtered backprojection reconstruction method and Hanning filters with a 0.33 cycles/pixel (H13) and 0.21 cycles/pixel (H20), cutoff frequency, respectively. The 3D studies were reconstructed using a Kinahan-Rogers [3] algorithm and also using H13 and H20 filters. The matrix size was 128 × 128 and the pixel size was 4.29 mm. Attenuation correction using an 8-min transmission scan was applied in all studies. In the 2D studies, Bergstrom [4] scatter correction was applied. For the 3D data, scatter correction was performed by fitting the tails of the sinogram to a 2D Gaussian [5]. Transaxial gated slices were transferred to GE Xeleris system for further gated analysis.

**Figure 1:**
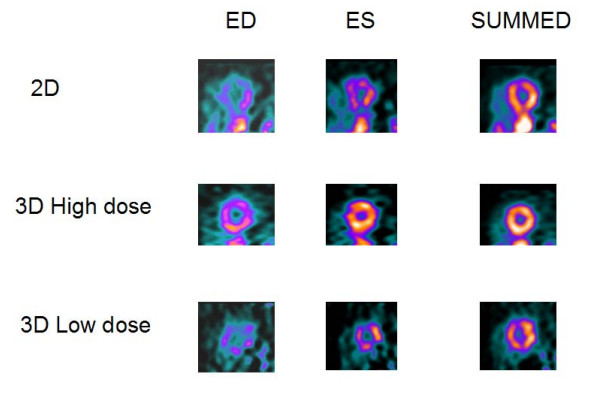
**Short-axis slices**. Mid-chamber short-axis slices in the 2D, 3D HD and 3D LD volunteer ^82^Rb gated myocardial PET study. Reconstruction filter was H13, i.e. Hanning filter with 0.21 cycles/pixel cutoff frequency.

**Figure 2:**
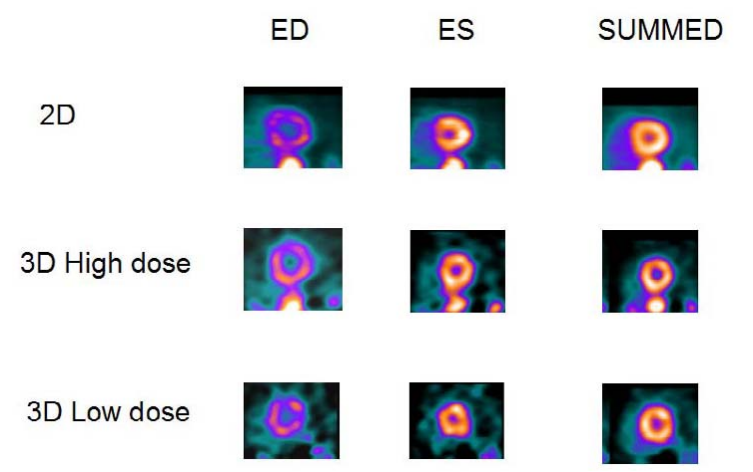
**Short-axis slices**. The same as on figure 1 but for H20, i.e. Hanning filter with 0.33 cycles/pixel cutoff frequency.

**Figure 3:**
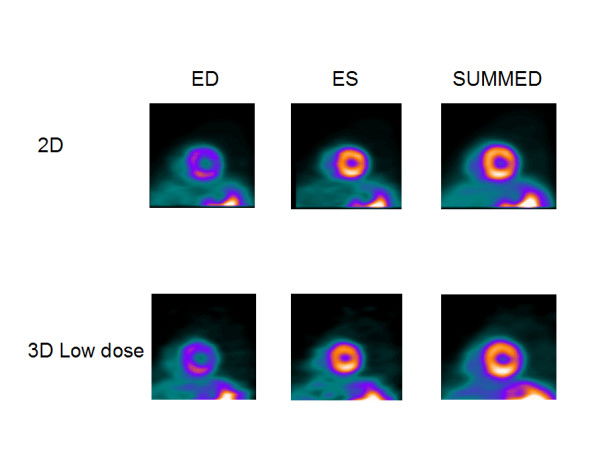
**Light volunteer**. Results of comparison for light (49 kg, 162 cm) volunteer, for 2D and 3D LD studies.

**Figure 4:**
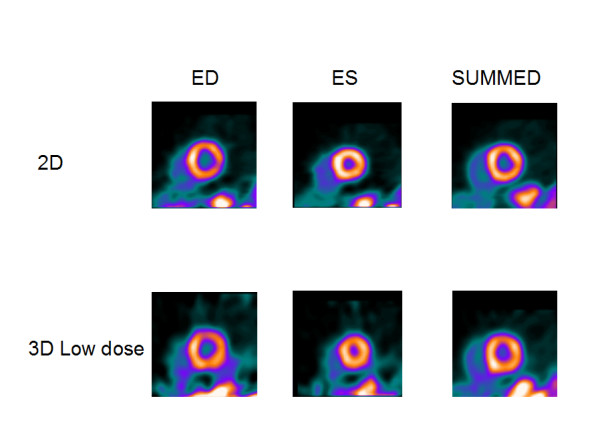
**Normal-weight volunteer**. Results of comparison for normal-weight (75 kg, 188 cm) volunteer, for 2D and 3D LD studies.

**Figure 5:**
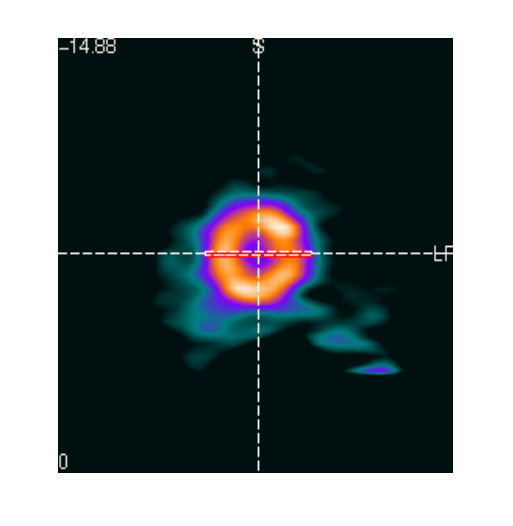
**Mid-chamber short-axis slices**. Mid-chamber short-axis slices profile ROI.

**Figure 6:**
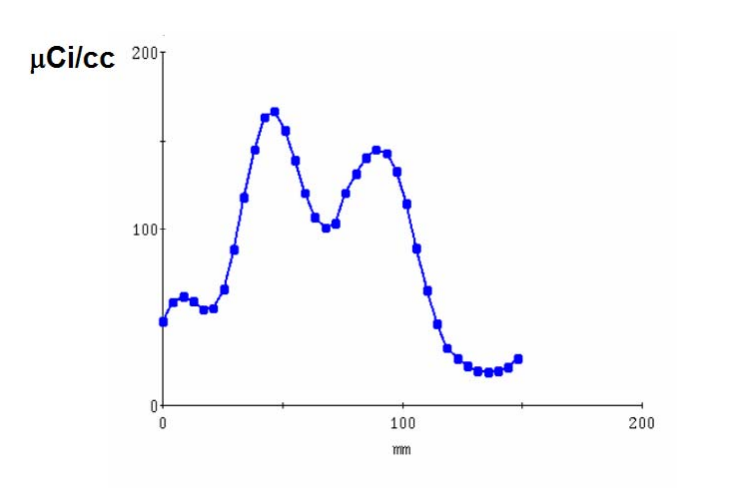
**Profile curve**. Corresponding profile curve from ROI shown in figure 5.

**Figure 7:**
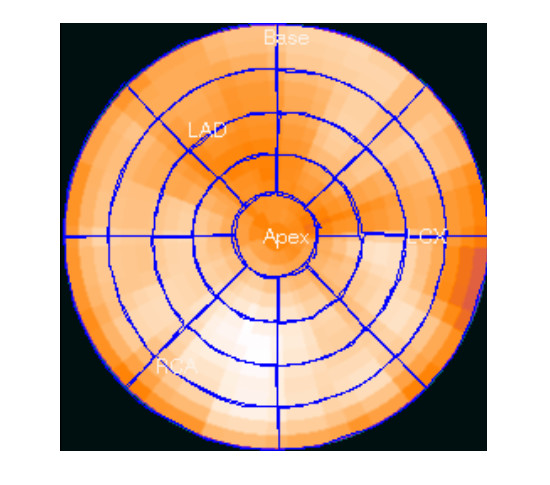
**Polar map**. Polar map and 33 segmental ROIs used in our comparison.

A mid-chamber short axis slice was used for analysis (Figs. 1, 2, 3, 4). In addition to end dyastoli (ED) and end systoli (ES) images, gated images summed over the cardiac cycles were also used in comparison. The contrast value, which was calculated as a ratio C = (A-B)/(A+B), where A and B are the average activities in the left ventricle (LV) and LV cavity respectively, was calculated from mid-chamber short-axis summed slice (Figs 5 and 6). The contrast values were used in comparison between 2D vs high dose 3D, 2D vs low dose 3D and high dose 3D vs low dose 3D studies, respectively. We used a paired t-test in the comparison of the contrast values. In our noise analysis, image noise was defined as the coefficient of variation (COV, 100 × SD/mean (%)). Summed short axis slices from apex to base were used to create circumferential profiles and polar maps on which we superimposed ROIs (Fig. 7), giving 33 segments. For each segment, the mean value and standard deviation was calculated.

Intrasegmental variance was investigated by calculating variance for each segment, as well as all segmental variances and the average coefficient of variance. F statistics were used to compare 2D and 3D HD studies, between 2D and 3D low dose studies and 3D HD and 3D low dose studies.

Intersegmental variance was investigated by applying a logarithmic transform on each mean segmental value and performing two-way ANOVA without replication. The effects of different patients, different segmental positions and interaction of different patients and segments (assumed none) on noise were tested. Again, F statistics were used to compare 2D and 3D HD studies, between 2D and 3D low dose studies and 3D HD and 3D low dose studies.

EFs were calculated using QGS program. Initially we did not plan to compare EF in 2D and 3D studies due to difference in the number of phases used to cover the heart cycle. As mentioned before, because of the dynamic memory limitations, only 5 phases were used in 3D gated studies. In retrospective comparison between 2D and 3D EFs, 3 sets of data were found to be corrupted and only 13 subject were used in the comparison. Also, our noise and contrast analysis showed that the 3D LD and 3D HD images were very comparable. Therefore, we did not distinguish between HD and LD 3D studies in the comparison between 2D and 3D EFs. A second reason for not splitting between HD and LD 3D studies was the limited number of subjects and we want to keep the same number of studies in 2D and 3D.

## Results

Figures 1 and 2 show the results for a 54-y-old, 183 cm, 90-kg man, who volunteered twice. In the first study, 3D imaging was performed with the high dose of 2220 MBq (60 mCi) of ^82^Rb. Three months later in a second study, 3D imaging was performed with the low dose of 740 MBq (20 mCi) of ^82^Rb. In both studies, 2D imaging was performed with the high dose of 2220 MBq (60 mCi) of ^82^Rb. Figures 1 and 2 show the 2D and 3D high (HD) and low (LD) dose cardiac short-axis slices at end-diastole, end-systole and summed over all phases, respectively. The figure 1 shows the results for sharper filter H13 with 0.33 cycles/pixel cutoff frequency, and figure 2 the same for the smoother filter H20 with 0.21 cycles/pixel cutoff frequency. The images in fig. 1 are quite noisy and routinely we decided to use smoother filters. For smoother filter H20, Fig. 2, the 3D HD images have slightly better contrast than 3D LD gated images, but both low and high dose 3D images are comparable in quality, that is, in contrast, scatter from adjunct activity and noise to the 2D images. Summed images are even more similar than end-diastole and end-systole images. In our other volunteers studies, 3D HD studies provided slightly better images, i.e., with less amount of noise and slightly better contrast, as summarized in Table 1 and table 2. However, the main goal of our project was to determine whether 3D low dose studies can replace the more expensive high dose 2D or 3D studies, providing images of comparable quality. Therefore, the next two clinical examples are comparing only 2D with 3D low dose studies. Figure 3 compares the 2D and 3D gated end-diastole and end-systole images, and summed over all phases images, in ^82^Rb PET imaging when the lower dose of ^82^Rb (740 MBq) was used in the 3D study. The subject is a 49-kg, 162 cm, 20-y-old female. Here, all corresponding images, i.e., 2D and 3D LD gated and summed are very similar. Only the images for the smoother H20 filter, which is routinely used, are shown. Figure 4 shows the results for a 75-kg, 188 cm, 32-year-old man. Again, in the 3D study the lower dose was used and only results for routine, H20 filter are given. The 2D and 3D gated and summed images are very alike.

**Table 1:** Comparison of contrast values for 2D and 3D high-dose (HD) and 3D low-dose (LD) in ^82^Rb PET myocardial imaging -smoother filter H20

	**2D**	**HD-3D**	**2D**	**LD-3D**
Contrast	0.33	0.38*	0.33	0.34
NAS × 10-4	3.64	3.70	3.85	5.58
NWS × 10-2	1.79	1.85***	1.82	1.91**

*p = 0.04 vs 3D LD, **p = 0.025 vs 2D and 3D HD, ***p = 0.02 vs 2D

NAS = noise among segments NWS = noise within segments

**Table 2:** Comparison of contrast values for 2D and 3D high-dose (HD) and 3D low-dose (LD) in ^82^Rb PET myocardial imaging – sharper filter H13

	**2D**	**HD-3D**	**2D**	**LD-3D**
Contrast	0.48	0.50	0.33	0.34
NAS × 10-4	4.43	5.17	8.18	9.18***
NWS × 10-2	2.00	2.00	2.00	2.00

***p < 0.001 vs HD

NAS = noise among segments NWS = noise within segments

Table 1 and table 2 gives the mean contrast values in mid-ventricular short-axis slice for 2D and 3D low and high dose studies for H20 and H13 reconstruction filters, respectively. The contrast values in 2D and 3D LD studies are very close. The contrast values in 2D and corresponding 3D HD studies are also close, with 3D HD studies having slightly higher values. The p value of 0.04 shows that there was no statistically significant correlation between noise in 3D low and high dose studies. The contrast values are higher for the H13 reconstruction filter than for the smoother H20 (table 1 and table 2). Noise among segments (NAS) and noise within segments (NWS) are also given for 2D and 3D low and high dose studies, for both filters. From table 1 and table 2 one can see that noise among and within the segments is higher for H13, i.e., for sharper filter than for H20 in 2D and 3D low and high dose studies. Noise among and within the segments were very similar for the 2D and 3D high dose (HD) studies, although there were no statistically significant correlations between noise within segments in 2D and 3D HD studies (p = 0.02). In comparison between 2D and 3D low dose (LD) studies, noise among and within the segments was moderately higher for the 3D LD studies. However, again there were no statistically significant correlations between noise among and within segments in 3D LD studies and 2D and 3D HD studies (p = 0.025 for H20 filter and p = 0.001 for H13 filter). For the sharper filter H13, noise within segments was higher than for the smoother filter H20 and was practically the same in 2D and 3D low and high dose studies. The H13 filter provided relatively high noise images and is not used routinely in clinical practice.

**Figure 8:**
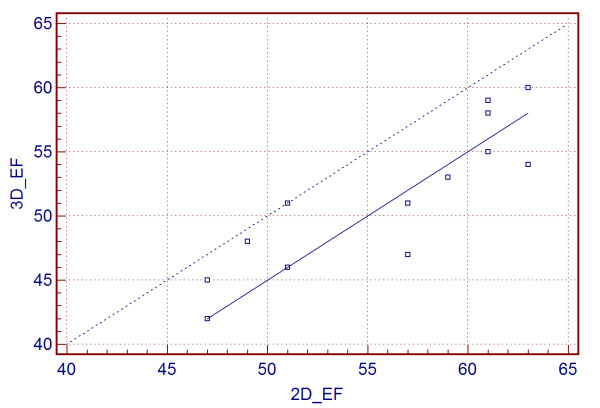
**Comparison between 2D and 3D ejection fractions**. Passing & Bablok regression scatter diagram with the regression line (solid line), the confidence interval for the regression line (dashed line) and identity line (x = y, dotted line), for the 2D and 3D EF. The correlation between 2D and 3D EF was 0.90 and there were no significant deviation from linearity (p > 0.10).

**Figure 9:**
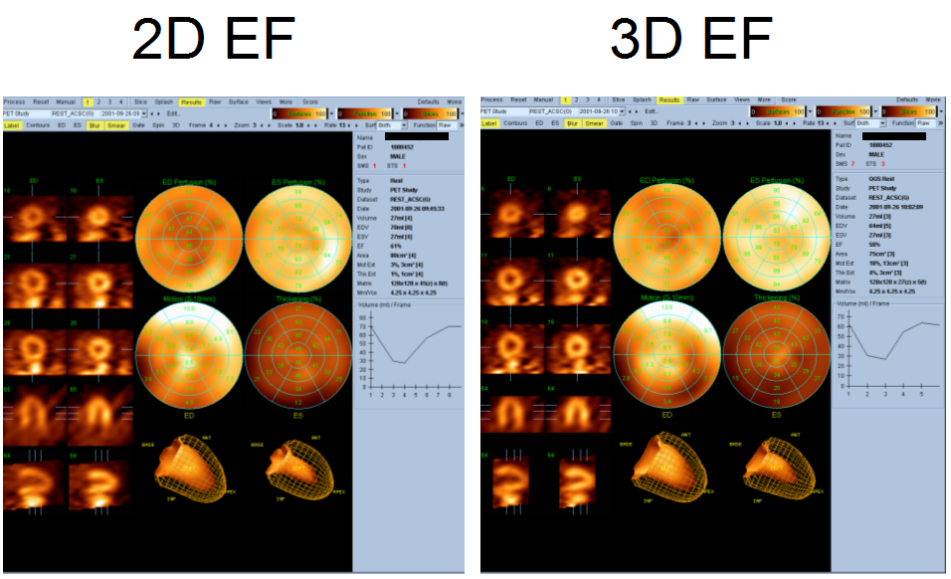
**Comparison between 2D and 3D ejection fractions of the same subject**. The 2D and 3D EF was 0.61% and 0.58 %, respectively.

The comparison between ejection fractions in 2D and 3D gated PET studies are shown in figure 8 and 9. There is a nice linear correlation between ejection fractions. The Pearson's correlation coefficient was 0.90 and there was no significant deviation from linearity (p > 0.10). The 2D ejection fractions were generally slightly higher but average difference from 13 subjects was 8.3%.

## Discussion

Gated myocardial images are usually count poor. Gated ^82^Rb PET myocardial imaging is an even greater challenge, because ^82^Rb has quite a short half-life, making ^82^Rb images count poor even without gating. Therefore, 3D gated ^82^Rb PET myocardial imaging has great appeal due to significantly higher sensitivity than 2D imaging. The first goal of our study was to investigate whether gated 3D high dose (2220 MBq) imaging, i.e., the same dose as in gated 2D images, would achieve better performance due to higher sensitivity. The results of our study indicate that gated 3D high dose images did not provide better images, because of the longer delay in acquisition from the time of injection in 3D HD imaging, as discussed below. The longer delay in 3D HD imaging diminishes the advantages of the higher sensitivity in 3D imaging in comparison with 2D imaging. The second question was to investigate the possibility of replacing high dose gated 2D and gated 3D imaging with gated 3D low dose (740 MBq) imaging. The advantage of 3D LD imaging, due to 3D mode higher sensitivity, is the same count rate as in 2D HD mode, but with a lower injected dose. This could lead to significant cost savings in the purchase of an ^82^Rb generator and thus could make myocardial ^82^Rb PET imaging more affordable [1].

Our results show that the contrast values between LV and LV cavity were practically the same in 2D and 3D low and high dose studies. However, the noise in 3D low dose studies has been slightly higher when compared with the 2D and 3D high dose studies. Nevertheless, in spite of the slight increase in noise in the 3D LD studies, the images are very comparable with high dose 2D and 3D images. Due to the fact that we used 8 phases in the 2D studies and only 5 phases in the 3D studies, the ejection fraction values in 3D studies were slightly underestimated, in average by 8.3%. The same effect was observed in the comparison between 16 and 8 phase gated SPECT studies, where the 8 phase studies show 3.71% lower ejection fractions [6].

Additional improvement in PET detectors [7] and better correction algorithms [8] can make the differences in contrast, ejection fractions and noise even smaller.

In terms of dosimetry, the effective dose equivalent for 2220 MBq (60mCi) of ^82^Rb is 2.66 mSv. The kidneys, as the critical organ, receives a dose of 19.98 mGy [9]. For 740 MBq (20 mCi) of ^82^Rb, the effective dose equivalent is 0.89 mSv with the kidneys receiving only 6.66 mGy, one-third of the dose for 2220 MBq (60mCi).

## Conclusion

On our dedicated high counting-rate performance PET system, 3D high dose (2220 MBq) gated PET imaging gives similar contrast and noise level as high dose 2D imaging. However, high dose 3D gated imaging did not achieve a better performance due to a necessary delay in acquisition from the time of injection, and slightly higher randoms and scatter fraction. Low dose (720 mBq) 3D gated imaging, while achieving similar contrast and ejection fractions, resulted in slightly higher noise, compared to either 2D or high dose 3D imaging. In view of these findings, we conclude that 3D low dose acquisition images with optimized filtering can probably give acceptable results with significant cost savings, related to purchasing an ^82^Rb generator, and considerable decrease in patient exposure.

## Competing interests

The author(s) declare that they have no competing interests.

## Authors' contributions

The authors, KK and JM, have made substantial contributions in acquisition, processing and analysing of data, and writing the manuscript. The JHK made significant contribution in calculating EF and analysing of data.

## Pre-publication history

The pre-publication history for this paper can be accessed here:


